# Assessment of a New E-Learning System on Thorax, Trachea, and Lung Ultrasound

**DOI:** 10.1155/2013/145361

**Published:** 2013-11-12

**Authors:** Colleen Cuca, Patrick Scheiermann, Dorothea Hempel, Gabriele Via, Armin Seibel, Magnus Barth, Tim O. Hirche, Felix Walcher, Raoul Breitkreutz

**Affiliations:** ^1^Ultrasound Regional Network SonoABCD and Frankfurter Institut für Notfallmedizin und Simulationstraining, Fachbereich Medizin der Johann Wolfgang Goethe-Universität, Klinikum der Johann Wolfgang Goethe-Universität, 60528 Frankfurt am Main, Germany; ^2^Klinik für Anaesthesiologie, Klinikum der Universität München, Campus Grosshadern, 81377 München, Germany; ^3^II. Medizinische Klinik und Poliklinik, Universitätsmedizin Mainz, 55131 Mainz, Germany; ^4^Anestesia e Rianimazione I, Fondazione IRCCS, Policlinico San Matteo, 27100 Pavia, Italy; ^5^Klinik für Anästhesiologie, Intensiv- und Notfallmedizin, Diakonie Klinikum Jung Stilling, 57074 Siegen, Germany; ^6^Fachbereich Pneumologie, Deutsche Klinik für Diagnostik GmbH, 65191 Wiesbaden, Germany; ^7^Klinik für Unfall-, Hand- und Wiederherstellungschirurgie, Klinikum der Johann Wolfgang Goethe-Universität, 60590 Frankfurt am Main, Germany; ^8^Zentrale Notaufnahme, Klinikum Frankfurt Höchst, 65929 Frankfurt am Main, Germany

## Abstract

*Background*. Lung ultrasound has become an emerging tool in acute and critical care medicine. Combined theoretical and hands-on training has been required to teach ultrasound diagnostics. Current computer technology allows for display, explanation, and animation of information in a remote-learning environment. *Objective*. Development and assessment of an e-learning program for lung ultrasound. *Methods*. An interactive online tutorial was created. A prospective learning success study was conducted with medical students using a multiple-choice test (Trial A). This e-learning program was used as preparation for a certified course followed by an evaluation of trained doctors (Trial B) by linear analogue scales. Pretests were compared with postcourse tests and sustainability tests as well as a posttest of a one-day custom classroom training. *Results*. In Trial A, during the learning success study (*n* = 29), the increase of correct answers was 11.7 to 17/20 in the post-test and to 16.6/20 in the sustainability test (relative change 45.1%, *P* < 0.0001). E-learning almost equalled scores of classroom-based training regarding gain and retention of factual knowledge. In Trial B, nineteen participating doctors found a 79.5% increase of knowledge (median, 95% CI: 69%; 88%). *Conclusion*. The basics of lung ultrasound can be taught in a highly effective manner using e-learning.

## 1. Introduction

The increasing demand for ultrasound applications in critical care medicine requires a discerning analysis of current and emerging training methods. In particular, ultrasound diagnostics of thorax, trachea, and lungs remain underutilized, although it has been shown to deliver more specific and sensitive results than chest X-ray, for example in cases of pulmonary edema regarding B-line differential diagnostics [1], pneumothorax [2–4], or pulmonary consolidation [5–8]. The safety and accuracy of ultrasound-guided interventions have similarly been demonstrated in critical care scenarios such as thoracocentesis [9, 10]. Pulmonary ultrasound is considered of utmost importance in critical care ultrasound curricula [11, 12] and encompasses the impartment of cognitive and psychomotor skills for accurate interpretation and acquisition of sonographic images.

Lecture- and seminar-based events, the current standards for ultrasound training, are often inaccessible due to time or financial constraints, posing a substantial hurdle to interested parties from medical students to board-certified doctors. While practical ultrasound skills may be quickly acquired in brief training courses [13, 14], the advancement of computer- and online-based training calls into question the necessity of a training seminar to convey pure theoretical knowledge of basic ultrasound concepts [15].

An online e-learning program can reduce the time, staff, and financial commitments of lecture-based training and promote active user involvement with the course material. The broad scope of e-learning and its accessibility enables knowledge to be conveyed quickly and effectively, ensuring its place in current and future learning applications [16]. Previous studies examining the effectiveness of e-learning programs in ultrasound training [17, 18] have primarily concentrated on using Internet media to present classical frontal lectures or enable a platform for tutoring, thereby removing the spatial drawback of attendance-based courses but retaining the typical teacher-student configuration. “Activation” of a learner is considered to be much more effective than passive listening to classical lectures [19].

We developed an interactive online training program as part of a critical care ultrasound training and aimed to analyze the success of independent theoretical skill development through an individual e-learning course as compared to a day-long training seminar with personal attendance provided by http://www.sonoabcd.de. http://www.sonoabcd.de is a network of multiple participating hospitals and institutions providing ultrasound education in Germany [13, 14, 20].

Our study was designed to determine the effectiveness and sustainability of content conveyed through the e-learning training program, as assessed by medical doctors and medical students. Further we compared factual knowledge gain with a generic attendance training.

## 2. Methods

### 2.1. Contents of the E-Learning System

The tutorial explains the basics of sonography and the most important physiological and pathological sonographic patterns of lung and pleura as defined by the International Consensus Conference on Lung Ultrasound [8]. Target groups were medical doctors and medical students. The module content is structured in 5 chapters: basics and pleural effusion, pneumothorax, pulmonary edema and consolidations, trachea, and workflow of a protocol-based lung ultrasound exam with exercises. Those 5 chapters contain a total of 21 units, which represent one screen each (Table 1).

### 2.2. Didactic Concept and Script

The theoretical approach was based on constructivism, which emphasizes a setting in which the learner arrives at his/her own conclusions and the “teacher” plays a more passive role in comparison to a classic frontal-lecture environment [21]. The script consists of 21 screens: one screen for each unit of the online tutorial. An important part in the learning process is interaction, whereupon the newly acquired knowledge is actively applied [22]. The units consist of ultrasound, X-ray and anatomical pictures, learning texts with key facts, and corresponding exercises: drag and drop, multiple choice, radio button, and fill-in-the-blank. An overview of the video clips and still images, including their respective sources, is provided (Table 1).

### 2.3. Production of the Online Tutorial

The anatomical sketches were drawn in coloured pencil and converted to Joint Photographic Experts Group (JPEG) standard format. All ultrasound videos were converted from their various formats to a flash-specific format using the Adobe Flash CS3 Video Encoder (Adobe Systems Inc., San Jose, CA, USA). The videos were cut in width and height as well as in duration to anonymize content as necessary and run in endless loops to facilitate uninterrupted viewing. Adobe Flash CS3 Version 9.0 was used as the main development tool, as its player plug-in allows presentation of videos and animations over the Internet in over 99% of browser programs installed in North America and Europe [23]. Each unit was saved as a source file (.fla), allowing later changes to be made, as well as an object file (.swf) for online display. Several learning units contain animations in terms of moving lines and arrows to denote specific structures in the sonographic videos. These animations were constructed by adding a layer for each object on the timeline on top of the videos, which creates the appearance of being inside or reaching into the videos.

The Web Kit Freiburg [24] was used as a template for the flash files. The Web Kit Freiburg itself was created in Flash (Adobe Systems Inc., San Jose, CA, USA) and delivered the framework and empty exercises originally created from the WEBGEO [25], in which a large number of the geography college courses were implemented as online tutorials. The user of the online tutorial loads an HTML file, which subsequently refers to all flash files containing the units. The online tutorial was first created in German language and later translated into English. The project's development, testing, and file sharing platform were hosted by the Basic Support for Cooperative Work (BSCW) Internet server of the Goethe-University of Frankfurt am Main, Germany [26].

### 2.4. Learning Success Study with Medical Students (Trial A)

**Figure 1:**
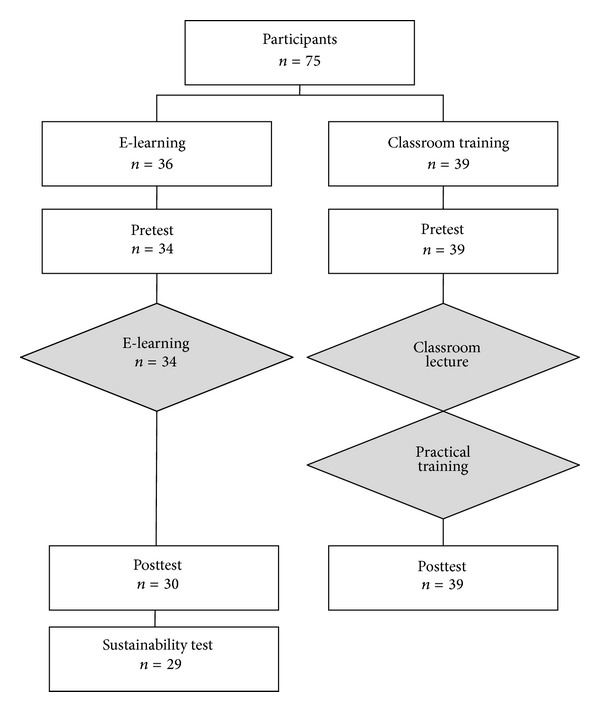
Flow diagram of all study participants. The classroom training cohort was part of the THOLUUSE study (with permission [14]).

A learning success study was conducted only with medical students. The entire study was done over the Internet, enabling every student to work through the online tutorial and take the tests at his own pace without any custom or hands-on training during the trial period. The online tutorial leveraged the WebCT platform [27] which offers the possibility of online testing and to which all students of Frankfurt University have access. The learning success study was composed of 20 multiple-choice questions with 4 answers each, out of which exactly one was correct. This test was copied from a former learning success study about a one-day course program with a combination of lecture and hands-on training entitled “Thorax and Lung Ultrasound in Emergencies/THOLUUSE [13, 14].” The participants of the THOLUUSE study were used as a reference group for comparison. Students took the test three times as pre-test, posttest, and sustainability test, with varied question and answer sequences. The pretest was available for one week, after which the online tutorial became available for two weeks. Following the tutorial, the posttest was administered within the 4th week, after which the online tutorial was no longer available for knowledge refreshment. After a two-week waiting period without access to the e-learning program, participants were allowed for one week at the seventh week of the study to take the sustainability test. At no time during the trial were the participants given access to the answer key. To augment participation compliance, ten monetary prizes between 20 and 50 Euros were distributed to the top scorers. According to the study design, only those students who participated in all three tests were incorporated into the final statistical consideration (Figure 1).

### 2.5. Evaluation of the Online Tutorial by Medical Students

The participants of the learning success study were subsequently asked to evaluate the online tutorial. This evaluation was carried out using the Internet tool survey monkey [35] and the questions were scaled discretely from 0 to 10 (Table 2). The participants were additionally asked about the time (in minutes) they spent working through the online tutorial.

### 2.6. Evaluation of the Online Tutorial by Medical Doctors (Trial B)

Four weeks prior to the one-day training courses on thorax, trachea, and lung sonography, the online tutorial was provided to medical doctors as a preparation for the course [13]. The participants received logins and passwords for the BSCW server. After their training course, they were asked to evaluate the online tutorial using a 13-question linear analogue self-assessment (LASA) survey. Each question was displayed individually on an A4 landscape-format page accompanied by a single, horizontal, 20 cm line without numbers. Participants could mark the line in between two extremes (example question as warm up “How was the coffee today?” ranging from “very bad” to “very good”). The marks were measured to an accuracy of 1 mm and those measurements converted into percent values (Table 3). The participating medical doctors were also given the opportunity to make additional comments on the second page of the survey.

### 2.7. Statistical Methods

Data analysis and sample size planning were performed using BIAS 9.04 (BIAS, epsilon Verlag, Frankfurt, Germany). Wilcoxon matched pairs test was used for analysis of pre- and postintervention test results. A *P* value of less than 0.05 was considered to be significant, thus indicating group differences. Distributions of variables are indicated as mean, median, and 25th/75th percentiles and shown as box plots. We aimed for descriptive explorative data analysis only. Case number calculation by a biostatistician control centre (Dr. H. Ackermann, Institute for Biostatistics and Mathematical Modeling, Hospital of the University of Frankfurt, Frankfurt am Main, Germany) revealed a minimum number of students completing all study parts of fifteen.

## 3. Results

### 3.1. Expert Evaluation

**Figure 2:**
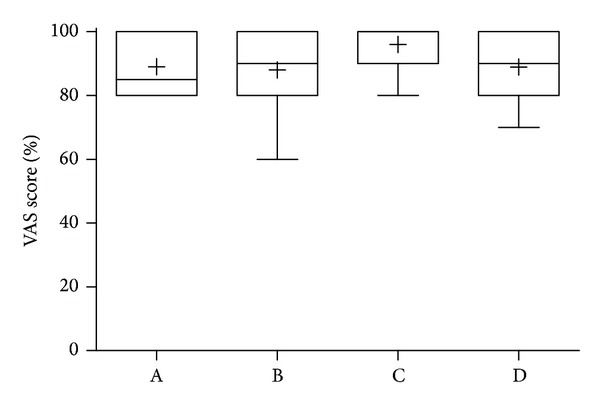
Evaluation of the online tutorials by lung ultrasound experts (*n* = 9, members of Volpicelli et al. [8]). Experts revised as peer group the e-learning by grading the content within a data sheet blinded to each other. Questions aimed to assess key questions: A: completeness? B: precision? C: sufficient as prelearning? D: would you use it at your own training programme?

Before the e-learning was used by our test group, an English version was evaluated by nine experts of the International Lung Ultrasound Consensus Conference [8]. This assured the quality of the tutorial prior to the start of testing and the conformity to consensus terminology. Thus, limitations in completeness and precision were improved, although not all of the 73 consensus statements were included as we did not want to integrate highly specialized lessons (e.g., ARDS) in the tutorial (Table 1). Remarkably, both main questions related to prelearning and use in their own training programmes were answered with a score of more than 90% (Figure 2).

### 3.2. Quantitative and Qualitative Evaluation by Students (Trial A)

#### 3.2.1. Quantitative Evaluation

**Figure 3:**
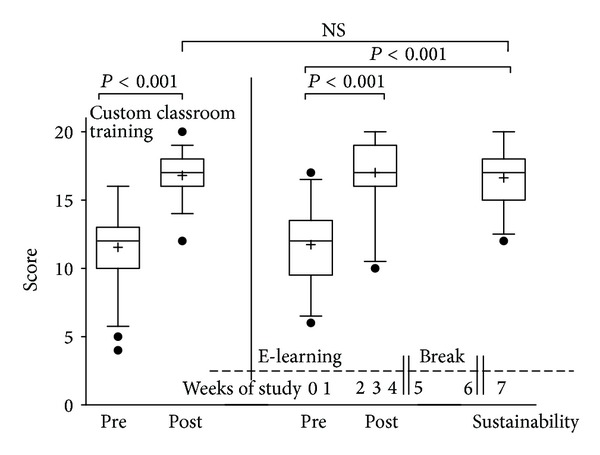
Comparison of two learning strategies: e-Learning versus custom classroom training. Absolute score indicates results of a pre- and posttest multiple choice questionnaire including 20 knowledge or image questions. Left of vertical line: results of learning success of a one-day presence only training on lung ultrasound “THOLUUSE” including 50% hands-on of (*n* = 54) medical doctors without E-Learning (taken from Breitkreutz et al. [14]). Right of dashed line: learning with e-learning but without custom classroom or hands-on training. E-Learning access was available within a learning phase of 4 weeks followed by a break without access of 2 weeks and completion with a sustainability test within the 7th week, results of *n* = 29 medical students. E-Learning was as effective as presence training regarding knowledge gain. (Boxslots contain median; line, box with 25/75 percentile, whiskers 5/95%, outliers, plus sign: mean), NS: not significant.

Of the 36 registered participants, 29 completed all three tests (81%), thus exceeding the required number (*n* = 15) determined by the study design. The participating students scored significantly higher in the posttest and sustainability test than they did in the pretest (Figure 3).

#### 3.2.2. Pretest versus Posttest

The mean score on the pretest was 11.7 (median 12, 25%; 75% percentile 9.5; 13.5,) while the mean of the posttest was 17.0 (median 17, 25%; 75% percentile 16; 19), equalling a relative improvement of 45.1% (*P* < 0.0001 in Wilcoxon's matched pairs test). Individually, 26 of 29 students displayed an improvement in score, two had a decline (which were also the worst scores in the posttest overall), and one had no change in score. Nearly half (14/29) of all participants achieved a score of 18/20 or higher (90%), suggesting a very positive learning effect (Figure 3).

#### 3.2.3. Pretest versus Sustainability Test

Similarly to that of the posttest, the sustainability test mean of 16.6 (median 17, 25%; 75% percentile 15; 18) was significantly higher than that of the pretest mean of 11.7 (median 12, 25%; 75% percentile 9.5; 13.5), displaying a relative improvement of 41.8% (*P* < 0.0001 in Wilcoxon's matched pairs test). Only one of the 29 participants had a lower score in the sustainability test as compared to the pretest (Figure 3).

#### 3.2.4. Posttest versus Sustainability Test

In comparison to the pre-test, both the posttest and sustainability test showed significant improvement. The results of the posttest 17 (median 17, 25%; 75% percentile 16; 19) and sustainability test 16.6 mean (median 17, 25%; 75% percentile 15; 18) did not differ significantly from each other (*P* = 0.237), suggesting a positive long-term learning effect amongst the participants of the e-learning lung ultrasound program (Figure 3).

#### 3.2.5. Comparison of E-Learning with THOLUUSE

Although the theoretical knowledge and learning success test were identical to that of the parent study, THOLUUSE [13] in which both theoretical and practical knowledge were conveyed in a day-long, attendance-based thorax and lung ultrasound course, it is important to acknowledge that the e-learning program is not able to incorporate the practical knowledge transfer that imparts the critical psychomotor skills required for ultrasound application. In addition to the identical multiple-choice test used, the results were also very similar in both studies. In both courses, the mean and median values of the pretest (e-learning mean: 11.7, median: 12, 25%; 75% percentile: 9.5; 13.5, THOLUUSE mean: 11.5, median: 12, 25%; 75% percentile: 10; 13) as well as the posttest (e-learning mean: 17, median: 17, 25%; 75% percentile: 16; 19, THOLUUSE mean: 16.8, median: 17, 25%; 75% percentile 16; 18) and sustainability test (e-learning median 17, 25%; 75% percentile 15; 18) were identical. The average relative improvement in both tests was also very similar (45.1% and 41.81% e-learning, 45.71% THOLUUSE) (Figure 3).

#### 3.2.6. Qualitative Evaluation of Medical Students

**Figure 4:**
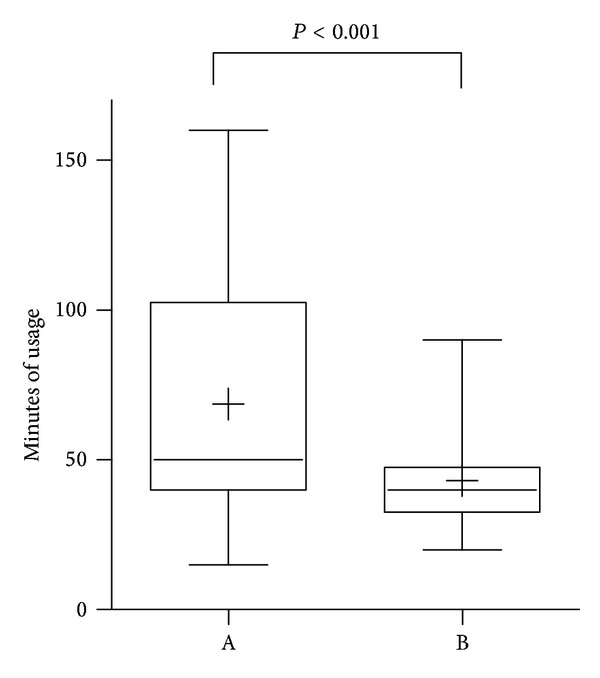
Self-evaluation of time spent with the online tutorial. A: *n* = 14; medical students after learning success study using survey monkey. B: *n* = 13; postgraduate medical doctors as part of the VAS questionnaires using paper-based questionnaires.

Of the 34 students who were asked to do so, 19 returned both a completed evaluation of the program and all tests they had participated in. In addition to 6 questions assessing the effectiveness of the program (Table 2), the students were also asked to estimate the time they took to complete the units. This question was answered by 14 of the 19 students and indicated that most (*n* = 8) users completed the program within a 40 to 60 minute time frame, corresponding to approximately 2-3 minutes per unit. Two students completed all units within 15–20 minutes, averaging less than 1 minute per unit, and three students took between 6 and 8 minutes per unit (Figure 4).

**Figure 5:**
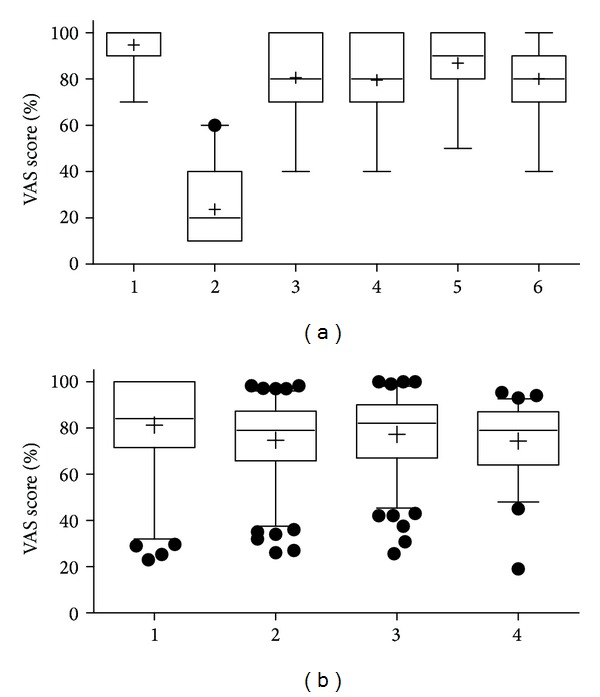
Self-evaluation at a discrete scale (0–10) of the online tutorial after completion, (a): of the learning success study by *n* = 19 medical students using Survey Monkey. 1: organization of the E-Learning, 2: own prior knowledge in lung ultrasound, 3: web-CT platform comfort, 4: own motivation, 5: general impression, 6: own learning success, (b): of *n* = 34 medical doctors after completion of preparation of the THOLUUSE training prelearning. Results of questions were grouped into areas of interest.1: personal effort, 2: scientific contents, 3: technical feasibility, 4: own learning success.

With the clear exception of question 2, the responses were overwhelmingly positive (Figure 4). The study organization was rated highest amongst the students (mean 90, median 100, 25%; 75% percentile 90; 100). The lowest rating, prior knowledge (mean: 20, median: 20, 25%; 75% percentile 10; 40), indicates that the majority of participants in the student-based study had no previous exposure to sonography. The rather high ratings of question 3 (mean: 80, median: 80, 25%; 75% percentile 70; 100) likely reflect the fact that many medical students in Frankfurt are familiar with the WebCT program and therefore had few or no problems operating it. The students rated their individual motivation predominantly positively (mean: 70, median: 80, 25%; 75% percentile 70; 100), corresponding to the positive self-assessment of motivation in the qualitative post-training survey of medical doctors. The e-learning program itself was rated second highest (mean: 80, median: 90, 25%; 75% percentile 80; 100), which likely indicates that many students have been exposed to similar pathways of online study and could use these for comparison. Personal assessment of individual learning success was also rated positively (mean: 80, median: 80, 25%; 75% percentile 70; 90), similar to the feedback of the identical question posed to the medical doctors (Figure 5).

### 3.3. Qualitative Evaluation by Medical Doctors (Trial B)

13 of the 34 completed surveys answered the question, “how long did the e-learning program take you in minutes?” Nine participants required between 30 and 50 minutes to complete the e-learning modules, two took more than 50 minutes, and two fewer than 30. Eleven doctors indicated that they had completed all answers in this reported time (Figure 5).

A total of 34 of 50 distributed surveys were returned with completed evaluations of the 13 questions using LASA.

The medians of each of the 13 questions surpassed 70. The response to question 10, “how significant was your knowledge gain?” (mean: 91, median: 100, 25%; 75% percentile 95; 100) was particularly positive (Figure 5).

The 13 individual questions were grouped into four categories: individual effort (Questions 9, 10, and 13), content evaluation (1, 3, 5, 12), practical application (2, 4, 7, 11), and success (6, 8) (Figure 5).

The category for individual effort received the highest ratings (mean: 79, median: 84, 25%; 75% percentile 72; 100), reflecting the fact that most of the doctors completed the entire program. Ratings were similarly high in the objective evaluations of program content (mean: 75, median: 79, 25%; 75% percentile 66; 87) and user-friendliness (mean: 77, median: 82, 25%; 75% percentile 67; 90) as well as in the subjective assessment of personal learning success (mean: 74, median: 79, 25%; 75% percentile 64; 87) (Figure 5).

The participating medical doctors were given the opportunity to make additional comments on the second page of the survey. Four doctors wrote that they were unable to complete the survey due to time constraints; another reported an unspecified error message, which prevented him from completing the program. Three participants in the first course commented that a user guide for the e-learning program would have been helpful; this was then assembled and distributed in the second course. Several positive comments were received from the participants regarding course and program organisation.

## 4. Discussion

Ultrasound is regarded to be a crucial diagnostic tool in many clinical questions of critical care medicine [36]. It is therefore imperative that ultrasound training is concisely and effectively conveyed to course participants. Appropriate application of ultrasound requires the ability to cognitively recognize and interpret pathological images as well as acquire the images using an ultrasonic device and psychomotor skills, the latter of which must be taught through practical hands-on training. Image recognition and theoretical basics, however, were demonstrated by our study to be effectively conveyed by an online e-learning program which requires neither the financial nor the time commitment that an attendance-based training demands. 

The knowledge gain in image recognition and basic sonographic theory is nearly identical to that of its parent study THOLUUSE [13, 14] which was able to demonstrate the effectiveness of a one-day classroom-based training (for thorax, trachea, and lung ultrasound). This strongly suggests that the theoretical portion of such a course could be completed at the participant's convenience previous to the practical, hands-on teaching.

In the evaluations received from the medical doctors, the median values of each of the 13 questions surpassed the score of 70%, enabling the conclusion that most participants were generally satisfied with the organization and content of the e-learning program.

The often-proven advantage of interactive learning in comparison to a lecture-based setting [37–39] is an additional argument for the application of e-learning programs to convey theoretical knowledge. Many previous e-learning studies have confined themselves to classical frontal lecture or student-teacher compositions [17, 18], thereby wasting the opportunity to exploit the advantage of an interactive learning system. The single study utilizing interactive e-learning in ultrasound [40] concerns procedural skills as opposed to the sonoanatomical knowledge the current study conveys. As critical as the psychomotor component of image acquisition is, it is mandatory to acquire the cognitive ability to recognize normal and pathological findings within the images [41]. The necessary interaction required by our e-learning program yielded the positive results that prove that the learned theoretical material is stored beyond the short-term memory of the participant, providing a solid base on which the practical skills of ultrasound diagnostics can be built.

Critics of e-learning question the effectiveness of such a program, which depends heavily not only on the competence of the learner, but also on his or her motivation to set learning goals and realize the steps to achieve them [42]. While motivation did not seem to be a hindrance within the boundaries of our study, further studies need to be carried out to compare actual (as opposed to self-estimated) performance on the practical portion of the training. A possible way to achieve this would be to merge the current study with the THOLUUSE study as a blended learning concept: offering the theoretical portion as e-learning and comparing the results of the subsequent practical training with that of THOLUUSE and as a future expanded concept adding further trainee-centred tools such as work books, quizzes, training in scenarios, and postcourse trainer/trainee interaction.

## 5. Limitations

Study limitations include the inability to randomize the study and the fact that participants were aware in advance of the intention and basic content of the e-learning program. Furthermore, participants were volunteers, which may have skewed motivation-related results in a more positive direction than if the modules had been completed by all students of a particular semester, for example. Several students seemed to speed through the e-learning program at an average rate of 1 minute per module, thus suggesting an overly rash preoccupation with their contents. The high mean scores of the posttest and sustainability test dispute this: despite the relatively quick processing of new knowledge, these inexperienced sonographers were able to apply their skills appropriately and effectively. Another limiting factor is that no sustainability test was performed by the medical doctors. A subsequent study would have to analyze both e-learning and classroom-based learning in a randomized fashion with both pretests and sustainability tests. 

## 6. Conclusions

E-learning has great potential to provide a substantial theoretical basis of sonographic principles and image recognition, the results of which are comparable to attendance-based courses. We recommend the use of e-learning to provide this knowledge to the widest audience possible, ensuring long-term retention of learned tenets and provoking interest in further practical training. E-learning is set to become a vital part of theoretical training in lung ultrasound and may induce future trainee-centred blended learning programs.

## Figures and Tables

**Table 1:** Source index of sonographic films and still images used in the online e-learning program modules.

No.	Image (Name)	Description	Source
1	Pleura anatomical sketch	Drawing of pleura, lung, recess, diaphragm, liver	M. Barth
1	Orientation film	Sonographic film of pleura, lung, recess, diaphragm, liver	T. Hirche
2	Artifact film	Sonographic film of physiological lung	R. Breitkreutz
3	Thorax photography	Photography with marked position of the probe	M. Barth
3	Curtain film	Sonographic film of the curtain phenomenon of the liver	R. Breitkreutz
4	Dorsal extinction film	Sonographic film of a rip over the lung	T. Hirche
4	Dorsal gain film	Sonographic film of blood vessels	T. Hirche
5	Pleural effusion film	Sonographic film of pleural effusion with atelectasis	T. Hirche
5	Pleural effusion in x-ray	X-ray of pleural effusion, right	T. Hirche
6	Reverberation film	Sonographic film of Reverberations	R. Breitkreutz
7	Seashore-Sign film	Sonographic film of a physiological lung in M-Mode	R. Breitkreutz
7	Bar-code-Sign film	Sonographic film of a pneumothorax in M-Mode	R. Breitkreutz
8	Pneumothorax	Sonographic film of a pneumothorax in B-Mode	R. Breitkreutz
8	Pneumothorax x-ray	X-ray of Pneumothorax, left	[28]
9	Lung point in B-mode	Sonographic film of lung point in B-mode	[29]
9	Lung point in M-mode	Sonographic image of lung point in M-mode	[30]
9	Lung point schematic sketch	Schematic explanation of the lung point	[30]
10	Lung pulse in M-mode	Sonographic film of lung pulse in M-mode	R. Breitkreutz
10	Lung pulse in B-mode	Sonographic film of lung pulse in B-mode with Doppler	T. Hirche
11	Trachea anatomical sketch	Drawing of the trachea in transversal sectional image	M. Barth
11	Trachea longitudinal sonogram	Sonographic longitudinal image of the trachea	R. Breitkreutz
12	Neck anatomical sketch transversal	Drawing of transversal sectional image of the neck	M. Barth
12	Trachea transversal sonogram	Sonographic transversal image of the trachea	R. Breitkreutz
13	Pneumothorax split screen	Sonographic image of a pneumothorax in split screen B-mode and M-mode	[30]
13	Lung point split screen	Sonographic image of the lung point in split screen B-mode and M-mode	[30]
14	Lung split screen	Sonographic film of physiological lung in split screen B-mode and M-mode	R. Breitkreutz
15	Thorax with probe	Thorax with ultrasound probe and 6 sectors for examination	R. Breitkreutz
15	Lung 6 split screens	6 images in split screen B-mode and M-mode of different sectors of the thorax, one of which with pneumothorax	R. Breitkreutz
16	Trachea longitudinal for practice	Sonographic longitudinal image of the trachea	R. Breitkreutz
16	Trachea transversal for practice	Sonographic transversal image of trachea	R. Breitkreutz
17	Pleural effusion film	Sonographic film of pleural effusion with atelectasis (as on screen No. 5)	T. Hirche
18	Alveolointerstitial syndrome	Sonographic film lung contusion, multiple B-lines	[29]
18	Lung consolidation	Sonographic film of consolidated lung parenchyma	R. Breitkreutz
18	peripheral parenchymal lesions	Sonographic image with multiple peripheral parenchymal lesions	[29]
18	peripheral parenchymal lesion	Sonographic image with multiple peripheral parenchymal lesion and B-line	[29]
19	Air bronchogram	Sonographic image of lunge with air bronchogram	[31]
20	Lung infarction	Sonographic image of lung infarction after pulmonary embolism	[31]
20	Triangular lung infarction	Sonographic image of triangular lung infarction after pulmonary embolism	[32]
20	Rounded lung infarction	Sonographic image of rounded lung infarction after pulmonary embolism	[32]
21	Pulmonary edema with 5 B-lines	Sonographic film of a pulmonary edema with 5 B-lines	[33]
21	Pulmonary edema with confluent B-lines	Sonographic film of a pulmonary edema with confluent B-lines	[34]

M. Barth, T. Hirche and R. Breitkreutz provided pictures from their private archives.

**Table 2:** Questions of qualitative evaluation survey of the e-learning program using the internet tool Survey Monkey. Questions were scaled discretely from 0 to 10.

The 6 questions regarding study and program setup and organization, personal initiative and learning success read as follows:”	
(1) How well was the study organized?	
(2) How good was your prior knowledge of sonography?	
(3) How suitable was WebCT as a framework for the e-learning program?	
(4) How high was your motivation for the e-learning program?	
(5) How good was your overall impression of the e-learning program?	
(6) How great was your learning success?	

**Table 3:** Questions of qualitative evaluation survey of the e-learning program using linear analogue self-assessment by medical doctors.

(1) How do you evaluate the subject matter?	
(2) How well were the modules defined?	
(3) Were enough details presented?	
(4) Could you recognize the structures on the ultrasound clips?	
(5) Was a central theme apparent throughout the modules?	
(6) How significant was your knowledge gain?	
(7) Was the time requirement acceptable?	
(8) How well prepared do you feel for the practical course?	
(9) How much time were you able to invest in the e-learning course?	
(10) How many units did you complete?	
(11) How well were you able to operate the e-learning program?	
(12) How solvable were the tasks in the e-Learning program?	
(13) How high was your motivation level for e-Learning?	
